# Development of a Guava Jelly Drink with Potential Antioxidant, Anti-Inflammation, Neurotransmitter, and Gut Microbiota Benefits

**DOI:** 10.3390/foods14132401

**Published:** 2025-07-07

**Authors:** Hai-Ha Nguyen, Jintanaporn Wattanathorn, Wipawee Thukham-Mee, Supaporn Muchimapura, Pongsatorn Paholpak

**Affiliations:** 1 Department of Physiology and Graduate School (Neuroscience Program), Faculty of Medicine, Khon Kaen University, Khon Kaen 40002, Thailand; nhaiha@ctump.edu.vn; 2 Department of Physiology, Faculty of Medicine, Khon Kaen University, Khon Kaen 40002, Thailand; meewep@gmail.com (W.T.-M.); supmuc@kku.ac.th (S.M.); 3 Research Institute for High Human Performance and Health Promotion, Khon Kaen University, Khon Kaen 40002, Thailand; 4 Department of Psychiatry, Faculty of Medicine, Khon Kaen University, Khon Kaen 40002, Thailand; ppaholpak@kku.ac.th

**Keywords:** guava, pomelo-derived dietary fiber, cognition, mental wellness

## Abstract

Due to the roles of oxidative stress, inflammation, and neurotransmitter imbalances in cognitive and mental dysfunction, we aimed to develop a functional drink with antioxidant and anti-inflammatory properties as well as the potential to support neurotransmitter balance for improved cognition and mental health. The Teng Mo, Fen Hong Mee, and Hong Chon Su guava varieties were screened for their polyphenol and flavonoid contents, antioxidant and anti-inflammatory effects, and suppressive effects on acetylcholinesterase (AChE), monoamine oxidase (MAO), GABA transaminase (GABA-T), and glutamate decarboxylase (GAD). Juice from the cultivar with the highest potential was selected and mixed with mint and honey syrups, pomelo-derived dietary fiber, ascorbic acid, agar, water, and fruit puree (pear/apple/orange) to create three guava jelly drink formulations. The formulation with pear puree showed the highest biological potential and was selected as the final product. It is rich in vitamin C, gallic acid, and dietary fiber, and provides approximately 37 Kcal/100 g. It also promotes the growth of lactic acid-producing bacteria in the culture. Thus, our drink shows the potential to reduce oxidative stress and inflammation, improve neurotransmitter regulation, and stimulate the gut–brain axis, thereby promoting cognition and mental wellness. However, clinical research is essential to confirm these potential benefits.

## 1. Introduction

Mental health disorders and cognitive deficits are currently regarded as major public health problems because they can exert profound effects on health-related quality of life (HQOL). It has been reported that one in every eight people worldwide live with a mental health disorder [1]. In addition, these conditions also result in high healthcare costs, which have been projected to reach approximately USD 6 trillion by 2030 [2]. Moreover, these disorders are the leading cause of global disability, and they account for approximately 48.5–60.0 million years lived with disability (YLD) [3]. Unfortunately, the therapeutic efficacy of most current medications against mental health disorders is still unsatisfactory and produces side effects [4]. To reduce this great socio-economic burden and provide effective prevention and health promotion effects, innovative and easily implementable strategies against mental health disorders should be considered.

Abundant scientific evidence reveals the link between diet and health benefits such as cognitive improvement and mental wellness. It has been revealed that foods that possess anti-inflammation and antioxidant properties decrease the risks associated with mental unwellness and cognitive impairment [5,6,7,8,9]. Both antioxidant and anti-inflammatory effects have been associated with many types of food, including macromolecules such as those present in whole grains, protein hydrolysates, and omega-3 fatty acids; micromolecules such as vitamin C, vitamin A, and vitamin E [5]; and phytonutrients such as polyphenols, phytosterols, and saponins [10]. Multiple lines of evidence have demonstrated that antioxidants and anti-inflammatory substances can improve mental health and cognitive function by modulating the brain’s plasticity via alterations to the survival, proliferation, and differentiation of brain cells [11]. Beyond the aforementioned antioxidant and anti-inflammatory effects on brain plasticity, the modulation of the gut–brain axis (GBA) can also affect cognition and mental health [12], via the modulation of brain plasticity or neurotransmission [13]. Based on the crucial roles of brain plasticity and neurotransmission induced by the stimulation of the GBA in cognitive function and mental health, the development of a functional drink containing ingredients that can modulate antioxidant and anti-inflammation properties and the GBA has gained attention.

*Psidium guajava* (guava), one of the most important commercial fruit crops in tropical and subtropical countries, belongs to the phylum Magnoliophyta, class Magnoliopsida, and family Myrtaceae. Guava fruit contains vitamins A and C, iron, calcium, and phosphorus. The content of vitamin C in guava fruit is higher than that in orange. In addition, it also contains saponins, oleanolic acid, arabopyranoside, lyxopyranoside, guaijavarin, quercetin, and flavonoids. Guava fruit possesses potent antioxidant activity, which appears to be associated with its vitamin C and polyphenol contents. It can decrease brain dysfunction and neurodegeneration [14]. In addition, it also possesses anti-inflammatory activity [15]. However, there are many cultivars of guava, and the contents of the potential substances as well as the biological activities mentioned earlier are varied [16]. Despite being a commercial fruit, guava has a short shelf life; therefore, its application as a natural resource for developing a functional drink may increase its value.

Pomelo (*Citrus grandis*) Osbeck, or *Citrus maxima* (Burm.) Merri—a plant in the Rutaceae family—is widely cultivated in Asia [17]. The fruit part of pomelo is frequently consumed, whereas the peel, which contributes around 30% of the fruit’s weight, is usually thrown away. This byproduct becomes waste and produces environmental pollution. However, pomelo peel is enriched in antioxidants, fiber, aromatic volatiles, and minerals [18]. Recent studies have demonstrated that pomelo peel-derived dietary fiber can improve dysbiosis and can increase the levels of both *Lactobacillus* and *Bifidobacterium* spp. [19], which can modulate the GBA, giving rise to improvements in cognition and mood disorders [20,21,22,23]. Owing to the beneficial effects of pomelo peel-derived dietary fiber (PPDF) on the GBA, which can improve cognition and mental health, it has been considered as an appropriate ingredient for developing functional drinks for improving cognition and mental health.

At present, it is well known that odor plays a major role in cognition and mood [24]. In addition, it also exerts an influence on eating behaviors [25]. To enhance flavor, mask undesirable tastes, and optimize the cognitive and mood regulation benefits, the cool and fresh aroma of mint (*Mentha cordifolia*)—a common culinary herb widely grown in Thailand [26]—has gained attention.

Most recent research on guava jelly has emphasized technological and sensory aspects, while its potential health benefits have received limited attention [27,28]. Owing to the demand for cognition and mental health promotion, especially among the middle-aged population, the fastest-growing market segment in the functional foods and beverages industry, this study aimed to develop a guava-based functional jelly drink containing PPDF and mint syrup, which was assessed for its potential cognition and mental health promotion effects; in addition, a sensory evaluation was performed in middle-aged volunteers.

## 2. Materials and Methods

### 2.1. Preparation of the Guava-Based Jelly Drink

Ripened fresh fruits of guava or Psidium guajava from 3 cultivars, including Teng Mo, Fen Hong Mee, and Hong Chon Su, were purchased between July and October 2024 from Tanaporn Garden, Nonthaburi Province, Thailand. In this study, the ripening stage of guava fruit was identified according to Khatun (2011) [29]. After a cleaning process, the fruit pulps were cut into small pieces, mixed with water (1 kg:1 L; *w*/*v*), blended by a Philips Blender HR2221/00 Series 5000 (Koninklijke Philips Electronics N.V., Amsterdam, The Netherlands), and boiled for 20 min. After boiling, the mixture was filtered using muslin cloth. The filtrate guava juice was used for the determination of the biological activities. In addition, guava juice was also used for preparing the guava juice jelly. The preparation of guava juice jelly was performed by using the modified method of Khatun [29]. Based on the crucial roles of oxidative stress, inflammation, and various neurotransmitters, including acetylcholine, monoamine, GABA, and glutamate, on cognition and mental health [11,30,31,32,33,34,35,36,37], the biological activities related to the mentioned factors were assessed, including antioxidant and anti-inflammation properties and the suppression effect on key enzymes in the inactivation processes of the mentioned transmitters. In brief, guava juice from the cultivar that demonstrated the highest potential to promote cognition and mental health (the highest antioxidant and anti-inflammation effects and the suppression effect on acetylcholinesterase, monoamine oxidase, GABA transaminase, and glutamic acid decarboxylase) was selected and mixed with various ingredients, shown in Table 1. After mixing, the mixture was boiled with constant stirring for 15 min or until it was dissolved. The measurement of the total soluble solids content, using a digital optical refractometer (HI 96801, Hanna Instruments, Woonsocket, RI, USA), was 8.4°Brix, and the measurements of pH via a pH meter (pH5 S, SANXIN Instrumentation, Shanghai, China) showed a value of 3.5. In addition, moisture was monitored by using a moisture analyzer (Nanjing Bonita Scientific Instrument Co., Ltd., Nanjing, China), indicating a content of 90.6%.

In this study, PPDF was prepared according to the previous method of Sanegthongpinit [38]. In brief, pomelo peels (Thong Dee Ban Thaen, Amphoe Ban Thaen, Thailand) were immersed in water for 2 h, boiled for 5 min, and dried in a hot air oven (Memmert M360, Memmert GmbH + Co. KG, Schwabach, Germany) at 45–50 °C for 24 h or until their moisture content was less than 10%. Then, it was milled with a multi-function high-speed disintegrator (Ezera 4500 A, Yongkang Hongtaiyang Electromechanical Co., Jinhua City, China) at a rotation rate of 35,000 rpm for 10 min. Following this step, the powder was sieved by a stainless steel sieve. The obtained PPDF was kept in dark packaging until used.

To prepare mint syrup, fresh leaves of *Mentha cordifolia,* or kitchen mint, were collected from Mueang Khon Kaen, Khon Kaen province, Thailand, during July–October 2024. They were cleaned, cut into small pieces, mixed with water at a ratio of 1:1 (*w*/*w*), and blended by a Philips Blender HR2221/00 Series 5000 (Koninklijke Philips Electronics N.V., Amsterdam, The Netherlands). After straining, the mint liquid was boiled with honey at a ratio of 1:1 (*w*/*w*) to obtain mint syrup. Honey sources were longan flowers harvested in the period of the longan blossom season, around February to March, from Suwan Farm Phueng Garden, Nong Bua Lamphu Province, Thailand.

**Figure 1:**
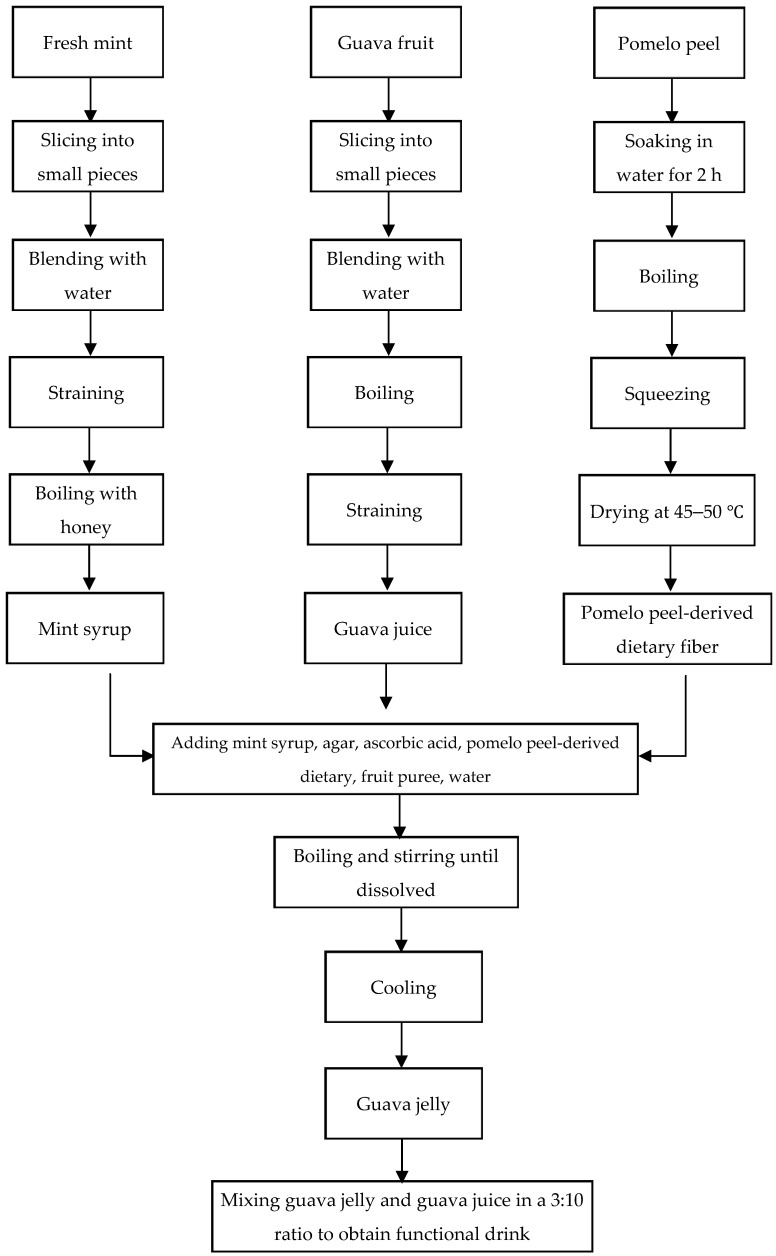
Schematic diagram of guava jelly drink preparation process.

The guava juice jelly drink was prepared by mixing guava juice and guava juice jelly at a ratio of 10:3 (*w*/*w*). After the preparation of three formulations (formulation 1—pear puree, formulation 2—apple puree, and formulation 3—orange puree), the best formulation was determined according to the assessment of biological activities and 9-point hedonic sensory scores. A hedonic rating test is a tool used to assess the level of satisfaction derived from consuming a food product, utilizing a 9-point hedonic scale from 1, indicating “extremely dislike”, to 9, indicating “extremely like” [39]. A placebo jelly drink was also prepared in parallel, except that no guava juice was used, and honey syrup was used instead of mint syrup, as shown in Table 1. To provide a better understanding, the whole process of the guava juice jelly drink preparation is explained with a schematic diagram in Figure 1.

### 2.2. Determination of the Profile of Flavonoids and Vitamin C Assessment

The profile of flavonoids of the developed jelly drink was determined by utilizing the HPLC system, consisting of a 515 HPLC pump and a 2998 Photodiode array detector (Waters Company, Milford, MA, USA). Chromatographic separation was conducted by using a Purospher^®^ STAR C-18 endcapped (5 μm), LiChroCART^®^ 250–4.6, and HPLC-Cartridge, Sorbet Lot No. HX255346 (Merck KGaA, Darmstadt, Germany). To generate an elution gradient, a mobile phase of 100% methanol (solvent A) (Fisher Scientific, Waltham, MA, USA) and 2.5% acetic acid (solvent B) (Fisher Scientific, Waltham, MA, USA) in deionized water was utilized. An aliquot of the tested substance at a volume of 20 μL was injected at a rate of 1 mL/min, and an elution gradient was carried out as described in the following: 0–17 min, 70% A; 18–20 min, 100% A; 20.5–25 min, 10% A. The profile was obtained at 280 nm, and Empower TM3 (Waters Company, MA, USA) was utilized to analyze the data [40].

Vitamin C assessment was outsourced to the Central Laboratory (Thailand) Co Ltd. (Bangkok, Thailand).

### 2.3. Determination of the Polyphenols and Flavonoids Contents, and Biological Activities

The contents of flavonoids and phenolic compounds of the guava juice, mint syrup, PPDF, and developed jelly drink were assessed in vitro. Antioxidant activity was evaluated by three different methods, including 2,2-diphenyl-1-picrylhydrazyl (DPPH), 2,2′-azinobis-3-ethylbenzothiazoline-6-sulfonic acid (ABTS), and ferric reducing antioxidant power (FRAP). In addition, the biological activities that are related to the pathophysiology of cognitive function and mood regulation, such as the suppression of acetylcholinesterase (AChE), monoamine oxidase (MAO), GABA transaminase (GABA-T), glutamate decarboxylase (GAD), and cyclo-oxygenase-2 (COX-2), were also determined [41,42,43,44,45]. To determine the stability of the developed jelly drink, all mentioned parameters were determined after the development and at 1, 3, and 6 months after the development, and stored in a dark bottle during a 6-month storage at 4 °C.

#### 2.3.1. Measurement of Phenolic Compounds

The reagents, with a 20 µL sample, 10 µL of 50% *v/v* Folin–Ciocalteu reagent (Sigma-Aldrich, St. Louis, MO, USA), and 140 µL of distilled water were mixed and incubated at room temperature for 8 min in a dark condition. Following this step, an aliquot of 20% sodium carbonate (Sigma-Aldrich, St. Louis, MO, USA) at a volume of 30 µL was added, and the mixture was subjected to a 2 h incubation period at room temperature under a dark condition. Finally, an absorbance at 765 nm was carried out by a microplate reader (Biochrom EZ Read 2000 Microplate Reader, Fisher Scientific, Waltham, MA, USA) [46]. Gallic acid (Sigma-Aldrich, St. Louis, MO, USA) at concentrations between 5 and 1000 µg/mL was used for the preparation of a standard calibration curve.

#### 2.3.2. Measurement of Total Flavonoids Content

The mixture containing a 100 µL sample and 100 µL of 2% methanolic aluminum chloride (Sigma-Aldrich, St. Louis, MO, USA) was prepared and subjected to a 30 min incubation period at room temperature. Finally, a measurement of the absorbance at 415 nm was carried out [47]. Quercetin (Sigma-Aldrich, St. Louis, MO, USA) at the concentration range of 5–1000 µg/mL was prepared as the standard calibration curve.

#### 2.3.3. DPPH (2,2-diphenyl-1-picrylhydrazyl) Assay

DPPH was applied to measure the ability to scavenge the stable free radical of the sample. A portion of 0.15 mM DPPH (Sigma-Aldrich, St. Louis, MO, USA) in 180 µL of ethanol was mixed with a 20 µL sample and incubated at room temperature for 30 min under a dark condition. Then, the absorbance at a wavelength of 490 nm was assessed [48]. The results were reported as an EC_50_ value, which is the effective concentration of the sample in mg/mL for scavenging 50% of free radicals.

#### 2.3.4. FRAP (Ferric Reducing Antioxidant Power) Assay

The FRAP method was carried out based on the capability to transform ferric tripyridyltriazine (Fe^3+^-TPTZ) to ferrous tripyridyltriazine (Fe^2+^-TPTZ). The FRAP solution, consisting of a mixture of 10 mM TPTZ (Sigma-Aldrich, St. Louis, MO, USA), 20 mM ferric chloride (Sigma-Aldrich, St. Louis, MO, USA), and 300 mM acetate buffer (Sigma-Aldrich, St. Louis, MO, USA) at a ratio of 1:1:10 was prepared. Then, the FRAP solution, at a volume of 190 µL, was mixed with a 10 µL sample. After mixing, the mixture was incubated at 37 °C for 10 min. Finally, an absorbance at 593 nm was measured [49].

#### 2.3.5. ABTS (2,2′-Azino-bis (3-ethylbenzothiazoline-6-sulfonic acid) Method

ABTS+ solution was prepared by mixing 2.45 mM potassium persulfate (K_2_S_2_O_8_) (Sigma-Aldrich, St. Louis, MO, USA) and 7 mM ABTS (Sigma-Aldrich, St. Louis, MO, USA) in a ratio of 3:2 (*v*/*v*). After mixing, the mixture was diluted with deionized water at a ratio of 1:20 before being used. Then, the mixture containing a 20 µL sample and 40 µL of distilled water was prepared. After adding 150 µL of ABTS+ solution, the absorbance at 750 nm was recorded [50]. The blank was prepared without a sample and replaced by ethanol. Trolox (Sigma-Aldrich, St. Louis, MO, USA) in the concentration range of 5–1000 µg/mL was prepared as the standard calibration curve for three methods of antioxidant activity evaluation.

#### 2.3.6. Determination of Acetylcholinesterase (AChE) Inhibition Activity

The colorimetric method was used to determine the AChE inhibition activity of the samples, as mentioned before by Ellman [51]. A reaction mixture, containing 10 µL of 0.2 M 5,5′-dithio-bis-2-nitrobenzoic acid (DTNB) (Sigma-Aldrich, St. Louis, MO, USA) and 200 µL of 0.2 M (pH 8.0) phosphate buffer saline (PBS), was placed under a 5 min incubation interval with a 10 µL sample at room temperature. Then, the recording of absorbance was assessed at the 415 nm wavelength before and after adding 10 µL of 30 mM acetyl thiocholine iodide (AChID) (Sigma-Aldrich, St. Louis, MO, USA). Donepezil (Sigma-Aldrich, St. Louis, MO, USA) at concentrations of 5–1000 µg/mL was used for the preparation of a standard calibration curve. The results were reported as EC_50_.

#### 2.3.7. Determination of Monoamine Oxidase (MAO) Inhibition Activity

The chromogenic solution was made from a combination of 1 mM vanillic acid (Sigma-Aldrich, St. Louis, MO, USA), 500 µM 4-aminoantipyrine (Sigma-Aldrich, St. Louis, MO, USA), and peroxidase (4 U/mL) (Sigma-Aldrich, St. Louis, MO, USA) in 0.2 M PBS (pH 7.6). The mixture, including a 25 µL sample, 50 µL of chromogenic solution, and 200 µL of 500 µM P-Tyramine (Sigma-Aldrich, St. Louis, MO, USA), was prepared and incubated at 37 °C for 30 min. The absorbance at 490 nm was recorded at the end of the incubation [52]. H_2_O_2_ (Sigma-Aldrich, St. Louis, MO, USA) at concentrations of 5–1000 µg/mL was used for the preparation of a standard calibration curve, and the results were reported as EC_50_.

#### 2.3.8. Determination of GABA-T Inhibition Activity

A sample aliquot at a volume of 50 μL was mixed with 200 μL of the mixture containing 20 mM GABA (Sigma-Aldrich, St. Louis, MO, USA), 10 mM α-ketoglutarate (Sigma-Aldrich, St. Louis, MO, USA), and 0.5 mM NAD (Sigma-Aldrich, St. Louis, MO, USA) in 0.05 M PBS pH 8.0. Then, the mixture was incubated at 30 °C for 30 min. At the end of the incubation period, the measurement of absorbance at 340 nm was carried out [53]. Vigabatrin (Sigma-Aldrich, St. Louis, MO, USA) at concentrations of 5–1000 µg/mL was used for the preparation of a standard calibration curve, and the results were reported as EC_50_.

#### 2.3.9. Determination of Glutamate Decarboxylase (GAD) Suppression Activity

GAD played an important role in the irreversible transformation of L-glutamic acid to GABA [54]. The determination of GAD suppression activity was performed by using the GAD antibody ELISA kit (MyBioSource, Inc., San Diego, CA, USA). The assessment was carried out according to the company’s guidelines. In brief, all reagents and standards were prepared according to the guidance. Then, the standard or sample was added to the reaction mixture, which was incubated at 37 °C for 90 min. At the end of the incubation period, 350 µL of washing lotion was added to each well and allowed to react for 30 s. Then, the well was shaken and cleaned with an absorbent paper. Following this process, rat GAD Ab antigen working solution was added to each well (100 μL for each). The reaction wells were sealed with adhesive tape and incubated at 37 °C for 60 min. After washing the plate 3 times, a conjugated enzyme working solution was added to each well (100 μL for each), and each was incubated at 37 °C for 30 min. At the end of the incubation, the plate was subjected to a 5-times washing process, and 100 µL of color reagent solution was added to each well, which was incubated at 37 °C for 30 min. Then, 100 µL of color reagent C was added to each well, mixed thoroughly, and the absorbance was read at 450 nm within 10 min. The standard curve was prepared from the dilution of rat GAD-Ab [55].

#### 2.3.10. Assessment of Cyclooxygenase-2 (COX-2) Suppression Activity

The chemical mixture, containing150 µL of 100 mM Tris-HCl buffer (pH 8.0), 10 µL of 5% heme (Cayman Chemical, Ann Arbor, MI, USA), a 10 µL sample, 10 µL of 1% COX-2 (Cayman Chemical, MI, USA), 20 µL of 100 μM arachidonic acid (Sigma-Aldrich, St. Louis, MO, USA), and 20 µL of 10 μM TMPD (N, N, N′, N′-Tetramethyl-p-phenylenediamine dihydrochloride) (Cayman Chemical, Ann Arbor, MI, USA), was prepared, and incubated at room temperature for 30 min. Then, an absorbance value was recorded at a wavelength of 590 nm [56]. Indomethacin (Sigma-Aldrich, St. Louis, MO, USA) at concentrations of 5–1000 µg/mL was used for the preparation of a standard calibration curve, and the results were reported as EC_50_.

### 2.4. Determination of Lactic Acid-Producing Bacteria (LAB)

After making a dilution series of samples, 0.1 mL dilutions of 10^−3^ to 10^−6^ were put onto a de Man–Rogosa–Sharpe (MRS) agar (HiMedia Laboratories LLC, Kennett Square, PA, USA) in an incubator for 48 h in an anaerobic environment. A colony-counting machine was utilized to determine the number of LAB [57].

### 2.5. Sensory Evaluation

A total of 60 Thai participants aged between 20 and 40 years (43.33% male and 56.57% female) assessed the sensory acceptability by using a 9-point hedonic scale [58,59]. The scale for the hedonic rating test is described as follows: 1 = dislike extremely, 2 = dislike very much, 3 = dislike moderately, 4 = dislike slightly, 5 = neither like nor dislike, 6 = like slightly, 7 = like moderately, 8 = like very much, and 9 = like extremely. The researcher explained both the tasting procedure and the evaluation process to the consumers prior to the test. All participants completed a consent form clarifying the test’s specific details before their participation. The space between the participants was appropriate to prevent them from exerting influence on each other. Three formulations of guava jelly drink were provided, with randomly labeled codes to ensure blinding. The consumers recorded the code of each formulation on a 9-point hedonic sensory questionnaire before evaluating it. Between each section of tests, participants had to rinse their mouths with water.

### 2.6. Statistical Analysis

Our study’s findings were all expressed as mean ± standard deviation (SD). The normality of the data was assessed using the Shapiro–Wilk test. Data that met the assumptions of normal distribution and homogeneity of variances were analyzed using one-way analysis of variance (ANOVA), followed by Tukey’s HSD post hoc test. For data that did not follow a normal distribution, the Kruskal–Wallis test was applied. *p* < 0.05 was used to examine the statistical significance. The data analysis was carried out by utilizing SPSS 26.0.

## 3. Results

### 3.1. Determination of the Polyphenols and Flavonoids Contents, and Biological Activities of Guava Juice, Mint Syrup, and PPDF

The results for polyphenol contents, antioxidant properties (DPPH, FRAP, ABTS), and enzyme activities related to neurotransmitters of mood and cognitive function (AChE inhibition, MAO inhibition, anti-GAD, GABA-T inhibition) of the three guava species and mint syrup are reported in Table 2 and Table 3. The results demonstrated that phenolic compounds ranged from 0.327 (unripe guava Hong Chon Su) to 0.658 mg GAE/g (ripe guava Fen Hong Mi), while flavonoid content ranged from 0.174 (unripe guava Teng Mo) to 0.192 mg Quercetin/g (ripe guava Hong Chon Su). Similarly, the results reported that phenolic compounds were 1.79 and 37.90 mg GAE/g, while flavonoids were 0.82 and 3.19 mg Quercetin/g in mint syrup and PPDF, respectively. The findings revealed that all species of guava juice, PPDF, and mint syrup exhibited antioxidant properties in three assays. The EC_50_ result exhibited that ripe Hong Chon Su (38.66 mg/mL) had the highest scavenging ability in the DPPH inhibition among the guava cultivars. In addition, unripe Fen Hong Mi (49.66 mg/mL) and ripe Fen Hong Mi (47.04 mg/mL) had the highest scavenging capacity in the FRAP and ABTS inhibition, respectively. The results demonstrated that the three types of guava juice, PPDF, and mint syrup also inhibited AChE and MAO. Unripe Fen Hong Mi guava juice had the highest AChE inhibitory effect (EC_50_ of 35.43 mg/mL) and MAO inhibitory effect (EC_50_ of 106.40 mg/mL), while the EC_50_ values were 24.68 and 98.40 mg/mL for mint syrup and the EC_50_ values were 1.57 and 11.23 mg/mL for PPDF, respectively. Regarding the GABA-T inhibition results, unripe Hong Chon Su had the highest activity (EC_50_ of 329.4 mg/mL) among the guava species, whereas the EC_50_ values were 267.75 and 5.73 mg/mL for mint syrup and PPDF, respectively. Finally, the lowest anti-GAD result among the guava types was from unripe Teng Mo, with 1.89 ng/mL, and its result was 0.32 ng/mL for mint syrup and 1.89 ng/mL for PPDF.

According to Table 2 and Table 3, the various cultivars of guava showed different strengths. To select the most suitable cultivar for developing guava jelly drink, the contents of polyphenols and flavonoids, and all biological activities, including antioxidant (via DPPH, FRAP and ABTS assays) and anti-inflammation (via the suppression effect of COX-2, a key enzyme in synthesis inflammatory mediator) effects, and the ability to modulate the change in neurotransmitter balance (via the suppression of AChE, MAO, GABA-T, and GAD) must be considered together. It was found that Fen Hong Mi guava juice at the unripe stage showed the highest potential to produce a positive modulation effect on cognition and mental health. Therefore, it was chosen for jelly drink development with PPDF and mint syrup because it showed high potential for neuroprotective, cognitive, and mood regulation effects.

### 3.2. Determination of the Polyphenol and Flavonoid Contents and Biological Activities of Three Formulations of Guava-Based Jelly Drink and Placebo

The results for flavonoids, phenolic compounds, antioxidant properties, and biological activities of three formulations and the placebo of jelly drinks are presented in Table 4. The three jelly drink formulations revealed significantly higher polyphenolic levels, antioxidant properties, and biological activities than the placebo. The results revealed that formulation 1 had the highest total phenolic compound (4.45 mg GAE/g) and flavonoid levels (0.69 mg Quercetin/g). The findings revealed that all the formulations of functional drinks exhibited antioxidant properties through three methods. The EC_50_ values revealed that formulation 1 (17.28 mg/mL) showed the highest scavenging ability among the three formulations via the ABTS method. However, formulation 2 (12.16 mg/mL) and formulation 3 (16.26 mg/mL) showed the highest scavenging capacities in the FRAP and DPPH methods, respectively. In addition, the results demonstrated that the three formulations also suppressed AChE and MAO activities. Formulation 1 showed the highest AChE and MAO suppression effects (17.21 and 71.42 mg/mL, respectively). Formulation 2 showed the lowest AChE suppression effect (32.86 mg/mL), while formulation 3 showed the lowest MAO suppression effect (94.16 mg/mL). According to the assessments of GABA-T and COX-2 suppression effects, it was found that formulation 3 revealed the most potent activities of GABA-T and COX-2, with EC_50_ values of 242.47 mg/mL and 115.16 mg/mL, respectively. Formulation 2 showed the lowest GAD suppression activity (1.23 ng/mL), whereas formulation 1 showed the highest GAD suppression activity (2.05 ng/mL). Taking all the data together, formulation 1 showed the highest potential to serve as a neuroprotective agent, cognitive enhancer, and mood regulator. In addition, Table 5 demonstrates the scores obtained from the sensory test performed by the potential customers via the hedonic scale. It was found that formulation 2 showed the highest overall acceptability score (7.00 ± 0.27), followed by formulation 3 (6.9 ± 0.27) and formulation 1 (6.47 ± 0.27), as shown in Table 5. However, the overall acceptability score failed to show a significant difference. Because formulation 1 had the highest health benefit potential, this formulation was selected for further study.

### 3.3. Determination of the Profile of Phenolic Compound, Flavonoid, and Nutrition Contents

**Figure 2:**
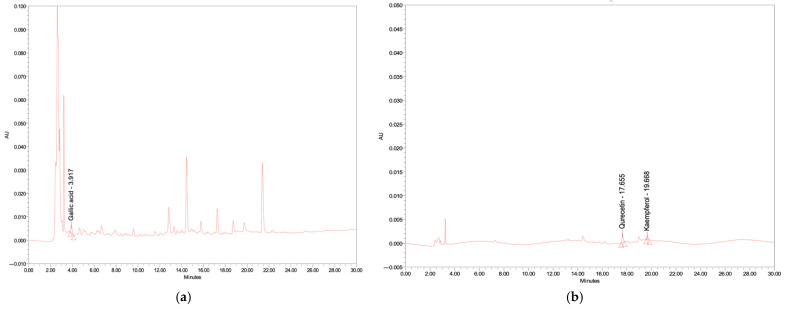
HPLC chromatograms of final functional drink at wavelengths of (**a**) 275 nm; (**b**) 370 nm.

The profile of flavonoids of the final functional beverage (formulation 1), shown in Figure 2, including gallic acid, quercetin, and kaempferol, present in the final product, was 0.6 ± 0.0, 0.1 ± 0.0, and 0.3 ± 0.0 mg/100 g of sample, respectively. The nutritional value of the final beverage was also determined by the Central Laboratory (Thailand) Co., Ltd., in Bangkok, Thailand. The final product was 100 g per serving; details of the nutritional value analysis, as well as determination methods, are shown in Table 6. In addition, the vitamin C content was also determined (Central Laboratory Co., Ltd., Bangkok, Thailand). It was found that the vitamin C content in the placebo was around 8.90 mg/100 g, whereas the vitamin C content in the selected formulation was 44.76 ± 0.0 mg/100 g.

### 3.4. The Shelf-Life Stability of Guava Jelly Drink Throughout the 6-Month Storage

Owing to the rapid degradation and instability of the polyphenols and flavonoids, we had to determine the stability of the product, or the functional beverage formulation 1, within a 6-month storage period. The changes in polyphenols, antioxidant properties, and biological activities of the jelly drink throughout the 6-month storage duration at 4 °C are presented in Table 7. It was found that the contents of both phenolic compounds and flavonoids decreased by less than 20% within a 6-month storage period at 4 °C. However, all biological activities related to neuroprotection, cognition, and mood regulation investigated in this study revealed changes of less than 20% only after a 3-month storage period. Most activities decreased by more than 20%, except GAD suppression activity, which showed a reduction of around 17% compared to baseline data (6 months). Therefore, a suitable storage time with good shelf-life activity was observed within a 3-month storage period. However, the sensory acceptability assessment by the potential customer can change during this storage period. Unfortunately, we did not determine the change in sensory acceptability together with the shelf life of the product; we considered only the biological activities and the possible active ingredient contents. This appears to be the weak point of this study because sensory acceptability is regarded as one factor that exerts an influence on the purchase intentions of customers [51]. In the future, this assessment should be explored together with the shelf-life assessment before moving the upscale process forward. In addition, after an upscale process, the determination of possible active contents and biological activities, together with sensory acceptability and shelf life, should also be assessed before moving forward to a clinical study.

### 3.5. Effect of the Developed Product on the Amount of LAB

To assess the prebiotic potential to modulate the gut–brain axis, we determined the amount of LAB in the product. It was found that the developed guava jelly drink revealed an amount of LAB of around 7.25 ± 1.35 × 10^6^, whereas no amount of LAB in the placebo was detected.

## 4. Discussion

The results of this study demonstrate that guava juice derived from unripe Fen Hong Mi guava has the highest potential for use in a functional beverage targeting neuroprotection, cognition, and mental health promotion. It was found that the highest polyphenol and flavonoid contents did not correspond to the most potent antioxidant activity, as assessed through DPPH, FRAP, and ABTS assays. Many studies have also demonstrated that phenolic compounds exhibit AChE [62,63,64], monoamine oxidase [65,66], GABA-T [64,67], and GAD [68,69] suppression effects. However, our findings revealed a lack of a close relationship between the aforementioned parameters and the polyphenol contents in various types of guava juice. A possible explanation for these findings may be associated with the interactions between various ingredients [70] in the guava juice.

After formulating various formulations of guava-based jelly drinks, the biological activities related to neuroprotection, cognitive enhancement, and mental wellness promotion, including antioxidant [71,72,73], anti-inflammation [8,74,75], and neurotransmitter regulation [76,77,78,79,80,81] activities, were also assessed. Our findings revealed that the guava jelly drink containing PPDF and pear puree showed the highest polyphenol and flavonoid contents. Although this formulation garnered a lower hedonic score, it showed the highest potential in terms of biological activities related to neuroprotection, cognition, and mental wellness, particularly mood regulation. In addition, within a 3-month storage period, the developed functional drink was quite stable. The degradation of functional ingredients such as polyphenols and flavonoids at 4 °C was less than 20%. Owing to these benefits, this formulation was selected as the final product.

Our findings also revealed that the developed functional drink contained high contents of vitamin C and polyphenolic compounds, particularly gallic acid, kaempferol, and quercetin. These results correspond with previous studies demonstrating the potential benefits of gallic acid [82,83], vitamin C [84,85], kaempferol [86,87,88,89], and quercetin [90,91,92,93] in terms of neuroprotection, cognition, and mental wellness. Moreover, the aforementioned substances also possess antioxidant and anti-inflammation properties [94,95,96,97]. Owing to the aforementioned properties and the biological activities observed in this study, we suggest that the observed biological activities in this study might be associated with the presence of the aforementioned substances. In addition, the interactions of various ingredients in the developed functional drink may also contribute to these roles.

In addition to the biological activities related to the physiological process of neuroprotection, cognition, and mental wellness, recent studies have also revealed that LAB could produce many substances that play important roles in gut–brain axis stimulation [98]. In this study, the developed functional drink could enhance LAB growth. Therefore, it is possible that following consumption, it could stimulate the growth of LAB, which, in turn, leads to gut–brain axis stimulation, supporting neuroprotection, cognition, and mental wellness [99,100,101,102]. Many studies conducted over the past decade have demonstrated that stimulation of the gut–brain axis can be induced by dietary fiber [103,104]. Based on this information, we suggest that—beyond vitamin C and the polyphenolic substances mentioned earlier—PPDF in the functional drink may also stimulate the gut–brain axis, resulting in the potential to improve neuroprotection, cognition, and mental wellness.

A strength of this study is the application of a multi-target drug design approach for the development of a functional drink. Since the regulation of cognition and mental wellness involves many factors and pathways, an effective approach must focus on multiple targets simultaneously. The data obtained from this study clearly reveal that the developed product can exert its potential action via many pathways, such as the suppression of oxidative stress and inflammation and the inactivation of various neurotransmitters with crucial roles in the regulation of cognition and mental health. In addition, it also showed the potential to increase LAB which, in turn, stimulate the gut–brain axis and may give rise to improvements in cognition and mental health. However, this requires further confirmation through clinical research. This study has several limitations. In addition to the lack of supporting clinical data, many points regarding the product should be considered before moving forward with clinical trials. Since this study was conducted at the laboratory scale, further evaluation is needed to assess the quality and stability of the product—including the required levels and stability of possible active ingredients, biological activities related to the physiological functions of cognition and mental health regulation, and the sensory acceptability of the product—after upscaling and storage. Moreover, the determination of contamination of all material resources, including guava, mint, and pomelo, should also be investigated before preparing the guava juice, mint syrup, and pomelo peel-derived dietary fiber to ensure safety from contamination. Due to budget limitations, we only determined the contamination of the final product, as it is essential for ensuring the safety of the product. The determination of probiotic bacteria changes after consumption, and the link between the change in gut microbiota and gut–brain stimulation, together with the improvements in cognition and mental health, should also be investigated in future research. Moreover, how changes in the oxidative stress status, inflammation, and neurotransmitters are pivotal in the regulation of cognition and mental function should also be explored, in order to facilitate a better understanding of the mechanisms driving the cognitive and mental health benefits of the proposed guava jelly drink.

## 5. Conclusions

The developed guava juice functional drink in this study is rich in polyphenolic compounds, particularly gallic acid, vitamin C, and PPDF. It exhibits antioxidant and anti-inflammation effects and suppression effects on AChE, MAO, GABA-T, and GAD. In addition, it also has the potential to promote LAB, thus stimulating the gut–brain axis. Based on its potential to modulate neuroprotective mechanisms—such as antioxidant and anti-inflammation effects, regulation of neurotransmitter balance through the suppression of key enzymes involved in neurotransmitter metabolism, and stimulation of the gut–brain axis—it may serve as a functional drink that promotes neuroprotection, cognition, and mental wellness. However, clinical research supporting these beneficial effects is still essential.

## Figures and Tables

**Table 1:** The ingredients of various formulations of guava jelly and placebo.

Ingredients	Placebo (%)	Formulation 1 (%)	Formulation 2 (%)	Formulation 3 (%)
Guava juice	0.00	63.55	63.55	63.55
Mint syrup	0.00	9.68	9.68	9.68
Honey syrup	9.68	0.00	0.00	0.00
Pomelo peel powder	0.14	0.14	0.14	0.14
Agar	0.55	0.55	0.55	0.55
Ascorbic acid	0.05	0.05	0.05	0.05
Fruit puree	5.45	5.45 (pear puree)	5.45 (apple puree)	5.45 (orange puree)
Water	84.13	20.58	20.58	20.58
Total	100.0	100.0	100.0	100.0

**Table 2:** The polyphenols, flavonoid contents, and antioxidant activities of 3 guava juice species, mint syrup, and PPDF.

Samples	Phenolic Compounds	Flavonoids	DPPH	FRAP	ABTS
	mg GAE/g	mg Quercetin/g	EC_50_ (mg/mL)		
Teng Mo (ripe)	0.598 ± 0.003 ***	0.186 ± 0.004	47.18 ± 0.87 ***	57.61 ± 0.88	79.78 ± 2.60 ***
Teng Mo (unripe)	0.609 ± 0.002 ***	0.174 ± 0.004 ***	52.05 ± 0.65 ***	69.02 ± 2.66 **	58.83 ± 0.59 ***
Fen Hong Mi (ripe)	0.658 ± 0.002 ***	0.189 ± 0.000	42.08 ± 1.09	89.07 ± 1.71 ***	47.04 ± 0.44 ***
Fen Hong Mi (unripe)	0.391 ± 0.004	0.188 ± 0.002	41.13 ± 0.70	49.66 ± 10.05	68.03 ± 2.40
Hong Chon Su (unripe)	0.327 ± 0.003 ***	0.185 ± 0.001	41.05 ± 0.77	79.13 ± 6.28 ***	56.36 ± 0.88 ***
Hong Chon Su (ripe)	0.341 ± 0.002 ***	0.192 ± 0.001	38.66 ± 0.37 *	60.37 ± 1.13	57.07 ± 1.00 ***
Mint syrup	1.79 ± 0.08	0.82 ± 0.08	99.40 ± 9.91	121.0 ± 2.08	102.68 ± 5.12
PPDF	37.90 ± 0.13	3.19 ± 0.42	5.9 ± 0.70	6.75 ± 0.65	2.21 ± 0.05
Standard	Gallic acid	Quercetin	Trolox 0.05 ± 0.00	Trolox	Trolox 0.05 ± 0.00

Data are reported as mean ± SD. *, **, *** *p* < 0.05, 0.01, 0.001 respectively; compared with unripe guava Fen Hong Mi.

**Table 4:** The polyphenol and flavonoid contents, antioxidant activities, and biological activities of the three formulations of jelly drinks and the placebo.

Parameters	Unit	Formulation 1	Formulation 2	Formulation 3	Placebo
Phenolic compounds	mg GAE/g	4.45 ± 0.08 ***	3.22 ± 0.13 ***	3.87 ± 0.27 ***	0.62 ± 0.19
Flavonoids	mg Quercetin/g	0.69 ± 0.04 ***	0.54 ± 0.02 ***	0.66 ± 0.04 ***	0.15 ± 0.03
DPPH	EC_50_ (mg/mL)	23.30 ± 1.09 ***	41.92 ± 1.78 ***	16.26 ± 1.18 ***	95.28 ± 1.35
FRAP		12.33 ± 0.67 ***	12.16 ± 0.74 ***	14.11 ± 1.85 ***	50.62 ± 3.01
ABTS		17.28 ± 0.15 ***	22.23 ± 0.55 ***	19.87± 1.18 ***	63.19 ± 3.77
AChE suppression effect		17.21 ± 1.62 ***	32.86 ± 1.62 **	30.5 ± 1.27 **	49.72 ± 7.97
MAO suppression effect		71.42 ± 3.92 ***	89.74 ± 5.97 **	94.16 ± 8.75 **	138.59 ± 16.54
GABA-T suppression effect		300.7 ± 8.32 ***	369.13 ± 11.38 ***	242.47 ± 7.27 ***	476.45 ± 18.68
COX-2 suppression effect		116.34 ± 11.83 ***	137.19 ± 6.63 ***	115.16 ± 10.90 ***	548.26 ± 7.07
GAD suppression effect	ng/mL	2.05 ± 0.05 ***	1.23 ± 0.11 ***	1.77 ± 0.02 ***	3.3 ± 0.18

Data are reported as mean ± SD. **, *** *p* < 0.01, 0.001 respectively; compared with placebo.

**Table 5:** The sensory hedonic score of three formulations of jelly drink (N = 60/group).

Formulation	Color	Smell	Taste	Appearance	Overall Acceptability
Formulation 1	6.43 ± 1.48	5.93 ± 1.74	6.43 ± 1.33	6.10 ± 1.65	6.47 ± 1.50
Formulation 2	6.47 ± 1.43	5.97 ± 1.90	6.87 ± 1.48	6.27 ± 1.66	7.00 ± 1.46
Formulation 3	6.47 ± 1.28	6.07 ± 1.72	7.20 ± 1.50	6.13 ± 1.36	6.90 ± 1.49
*p*-value	*p* = 0.956	*p* = 0.995	*p* = 0.085	*p* = 0.824	*p* = 0.378

Data are reported as mean ± SD.

**Table 6:** The nutritional values for 100 g of functional jelly drink.

Test Item	Result	Reference Method
Calories	37.70 Kcal	In-house method TE-CH-169 based on *Method of Analysis for Nutrition Labeling* (1993) P. 106. [60]
Carbohydrate	8.72 g	In-house method TE-CH-169 based on *Method of Analysis for Nutrition Labeling* (1993) P.106. [60]
Cholesterol	Not Detected	In-house method TE-CH-143 based on AOAC (2019) 994.10. [61]
Fat	0.18 g	AOAC (2019) 922.06. [61]
Protein	0.30 g	AOAC (2019) 981.10. [61]
Total dietary fiber	0.28 g	In-house method TE-CH-076 based on AOAC (2019) 985.29. [61]
Total sugar (HPLC)	6.87 g	In-house method TE-CH-164 based on AOAC (2019) 977.20. [61]

**Table 7:** The shelf-life stability of polyphenol and flavonoid contents, antioxidant activities, and biological activities of the functional drink throughout the 6-month storage period at 4 °C.

Parameter	Units	Baseline	1 Month	3 Months	6 Months
Phenolic compounds	μg GAE/mg	4.45 ± 0.08	4.43 ± 0.20	4.42 ± 0.26	3.95 ± 0.19 *
	% change		−0.45	−0.67	−11.24
Flavonoids	μg Quercetin/mg	0.69 ± 0.04	0.71 ± 0.09	0.70 ± 0.10	0.68 ± 0.07
	% change		2.82	1.43	−1.45
DPPH	EC_50_ (mg/mL)	23.3 ± 1.09	22.78 ± 1.03	24.47 ± 0.97	30.45 ± 1.51 ***
	% change		2.23	−4.78	−23.48
FRAP	EC_50_ (mg/mL)	12.33 ± 0.67	11.87 ± 0.19	13.01 ± 0.37	17.37 ± 2.38 **
	% change		3.73	−5.23	−29.02
ABTS	EC_50_ (mg/mL)	17.28 ± 0.15	17.65 ± 0.94	18.04 ± 1.48	32.86 ± 0.55 ***
	% change		−2.10	−4.21	−47.41
AChE suppression	EC_50_ (mg/mL)	17.21 ± 1.62	16.45 ± 0.99	20.94 ± 1.40 *	46.20 ± 0.81 ***
	% change		4.62	−17.81	−62.75
MAO suppression	EC_50_ (mg/mL)	71.42 ± 3.92	73.08 ± 1.50	83.55 ± 3.48 *	86.10 ± 3.93 **
	% change		−2.27	−14.52	−17.05
GABA-T suppression	EC_50_ (mg/mL)	300.70 ± 8.32	304.90 ± 5.59	308.08 ± 3.86	468.59 ± 41.79 ***
	% change		−1.38	−2.40	−35.83
GAD suppression	ng/mL	2.05 ± 0.05	1.95 ± 0.05	2.32 ± 0.04 **	2.47 ± 0.03 ***
	% change		4.91	−11.47	−16.81
COX−2 suppression	EC_50_ (mg/mL)	116.34 ± 11.83	110.83 ± 3.34	131.79 ± 35.78	153.53 ± 10.00
	% change		4.74	−11.72	−24.22

Data are reported as mean ± SD. *, **, *** *p* < 0.05, 0.01, 0.001, respectively; compared with first day of storage at 4 °C.

**Table 3:** The biological activities of three guava juice species, mint syrup, and PPDF.

Samples	AChE Inhibition	MAO Inhibition	GABA-T Inhibition	Anti-GAD
	EC50 (mg/mL)			ng/mL
Teng Mo (ripe)	51.74 ± 6.31	121.63 ± 2.93	388.63 ± 26.10	2.05 ± 0.01 ***
Teng Mo (unripe)	96.78 ± 4.13 ***	138.31 ± 6.44 **	554.00 ± 84.42 ***	1.89 ± 0.01 ***
Fen Hong Mi (ripe)	67.19 ± 5.17 **	152.59 ± 14.65 ***	585.63 ± 32.30 ***	2.65 ± 0.03 ***
Fen Hong Mi (unripe)	35.43 ± 5.31	106.4 ± 2.19	348.12 ± 21.12	4.21 ± 0.01
Hong Chon Su (unripe)	63.91 ± 12.8 **	152.09 ± 5.42 ***	329.40 ± 20.12	3.92 ± 0.01 ***
Hong Chon Su (ripe)	41.09 ± 2.79	166.02 ± 4.02 ***	380.48 ± 8.16	3.61 ± 0.02 ***
Mint syrup	24.68 ± 4.05	98.40 ± 5.37	267.75 ± 9.30	0.32 ± 0.13
PPDF	1.57 ± 0.35	11.23 ± 2.50	5.73 ± 0.24	1.89 ± 0.03
Standard	Donepezil 0.18 ± 0.01	H_2_O_2_ 0.09 ± 0.00	Vigabatrin 0.80 ± 0.01	GAD-Ab

Data are reported as mean ± SD. **, *** *p* < 0.01, 0.001 respectively; compared with unripe guava Fen Hong Mi.

## Data Availability

The data presented in this study are available on request from the corresponding author. The data are not publicly available due to trade secrecy and the ongoing patent registration process.
